# Effects of repetitive transcranial magnetic stimulation in the treatment of attention-deficit hyperactivity disorser: A case study

**DOI:** 10.1192/j.eurpsy.2021.1317

**Published:** 2021-08-13

**Authors:** E. Asimoglou, A. Tsakiri, P. Gkikas, S. Kalimeris, G. Karampoutakis

**Affiliations:** Psychiatric And Neurological Center, SMART CNS CENTER, ATHENS, Greece

**Keywords:** rTMS, Attention- Deficit Hyperactivity Disorder

## Abstract

**Introduction:**

Although there are very effective treatment approaches for Attention Deficit Hyperactivity Disorder (ADHD) available, the clinical management has its limits making new treatment modalities a necessity. Evidence suggests that low frequency repetitive transcranial magnetic stimulation (rTMS), improves hyperactivity, impulsivity, and attention deficit of individuals with ADHD.

**Objectives:**

The aim of this case study is to present the effectiveness of rTMS on hyperactivity, impulsivity, and attention deficit of an individual with ADHD.

**Methods:**

This is a case study of a 22 year old male diagnosed with ADHD. The protocol applied was 2 weeks of daily rTMS sessions. This involved repetitive TMS to the right dorsomedial prefrontal cortex (10 Hz, 3.000 pulses, 120% motor threshold) to treat attention deficit, hyperactivity and impulsivity. Assessments were conducted using Adult ADHD Questionnaires, the Jasper and Goldberg questionnaire and WHO self- reported scale at baseline, 1 month and up to 3 months follow up.

**Results:**

The patient showed overall improvement in scores in both ADHD scales with scores dropping more than 50 % in both scales from pre treatment to 3 months follow up. No side effects were recorded during therapy

**Conclusions:**

Our findings suggest that the use of rTMS therapy in individuals with ADHD, is an efficacious and safe therapeutic treatment option.

